# Culturable rare actinomycetes from Indian forest soils: Molecular and physicochemical screening for biosynthetic genes

**Published:** 2018-04

**Authors:** Sunita Bundale, Jaya Singh, Deovrat Begde, Nandita Nashikkar, Avinash Upadhyay

**Affiliations:** 1 Hislop School of Biotechnology, Hislop College, Nagpur, Maharashtra, India; 2 Department of Biochemistry, Dr. Ambedkar College, Deeksha bhoomi, Nagpur, Maharashtra, India

**Keywords:** Rare actinomycetes, Polyketide synthases, Non ribosomal peptide synthetases

## Abstract

**Background and Objectives:**

Rare actinomycetes are a promising source of novel metabolites of pharmaceutical importance. The current study focussed on selective isolation of specific genera of rare actinomycetes and screening the isolates for biosynthetic genes particularly polyketide synthases (PKS) and non ribosomal peptide synthetases (NRPS).

**Materials and Methods:**

The soil samples were subjected to various pre-treatments like 1.5% phenol treatment, 0.3% chloramine T treatment, benzethonium chloride treatment, etc. and plated on selective media supplemented with specific antibiotics targeting rare genera of actinomycetes. The putative rare actinomycete isolates were screened for bioactivity using agar cross streak method and agar well diffusion method. The ability of the isolates to produce anti-quorum sensing compounds was tested against *Serratia marcescens*. The isolates were also screened for the presence of biosynthetic gene clusters associated with PKS-I, PKS-II and NRPS pathways using the degenerate primer sets K1F-M6R, KSα/KSβ and A3FA7R, respectively. The expression of these gene clusters was tracked by physicochemical screening of the extracts of isolates using spectroscopic and chromatographic techniques.

**Results:**

In this study, 1.5% phenol treatment was found to be the most promising followed by heat treatment and chloramine treatment. Our studies showed that ISP5 agar was the best for isolation of rare genera followed by ISP7, Starch Caesin agar and ISP2 supplemented with antibiotics like gentamicin, nalidixic acid and streptomycin. *Micromonospora* was the most abundant genus followed by *Dactylosporangium. Actinomadura, Nocardiopsis* and *Actinoplanes* were almost equal in number. Primary screening showed that 92% of the isolates were active against one of the test organisms. Thirty seven isolates were found to produce anti-quorum sensing (QS) compounds. NRPS sequences were detected in thirty nine isolates (42.8%), whereas PKS-I and PKS-II sequences were detected in seventeen and twenty eight strains (18.6% and 30.7%), respectively.

**Conclusion:**

Nine type I and type II polyketide-producing isolates as well as six peptide-producing isolates were found. The peptide extract of isolate KCR3 and a polyketide extract of isolate NCD10 were found to possess anti-tumor activity exhibiting an IC_50_ value of 3 μg/ml and 2.5 μg/ml against HeLa cells.

## INTRODUCTION

Actinomycetes have a proven capacity to produce novel antibiotics, and many screening programs have been carried out leading to the discovery of numerous new bioactive molecules, which have subsequently found their way into various clinical uses ranging from control of infections to cancer treatment (1). However, the chances of discovering commercially potent secondary metabolites from well-known actinomycetes is becoming increasingly difficult due to the practice of wasteful screening that is leading to rediscovery of the known bioactive compounds (2). This emphasizes the need to isolate and screen the undiscovered representatives of the less explored actinomycetes. Recent evidence has demonstrated that rare actinomycete species, which are often very difficult to isolate and cultivate, might represent a unique source of novel biologically active compounds (3) and methods designed to isolate and identify a wide variety of such actinomycetes have been developed (4). These methods include a variety of pretreatment techniques in combination with appropriately supplementing selective agar media with specific antimicrobial agents.

Although bioactivity guided screening is the oldest and most common screening strategy, in the recent years, PCR based approaches have been used successfully to amplify genes associated with secondary metabolite biosynthesis (5). Polyketide synthases and non-ribosomal peptide synthetases are the major enzymes of secondary metabolite synthesis. A few examples of classes of antibiotics produced through these biosynthetic pathways include ansamycins, tetracyclines, polyenes and glycopeptides (6). In the present study, degenerate primer sets were used to screen for the presence of biosynthetic gene clusters associated with PKS-I, PKS-II and NRPS pathways in genomic DNA of one hundred and seventeen rare actinomycete strains.

Though the presence of the biosynthetic genes in isolates can be proved by molecular screening, it can not confirm the production of the compounds. Hence, expression of these gene clusters was tracked by physicochemical screening of the isolates using spectroscopic and chromatographic techniques for the analysis of crude extracts and prediction of the bioactive compounds. Such studies have been carried out previously on *Streptomyces*, a genus of actinomycetes (7, 8). However, to the best of our knowledge, these methods have not been used for screening non-*Streptomyces* rare actinomycetes, the focus of this study.

Research on actinobacteria is still in its infancy in India. Hence the aim of the present study was to determine the diversity of culturable bioactive rare actinomycetes from Indian forest soils and screen them for the presence of biosynthetic genes using PCR. The metabolic profile and anticancer activity of potent and promising isolates was also studied.

## MATERIALS AND METHODS

### Test organisms and cell lines.

The target strains used for screening antimicrobial activity were procured from microbial type culture collection and gene bank (IMTECH, Chandigarh, India) and were: *Micrococcus luteus* MTCC 106, *Bacillus subtilis* MTCC 441, *Escherichia coli* MTCC 443, *Pseudomonas aeruginosa* MTCC 741, *Serratia marcescens* MTCC 4822, *Candida albicans* MTCC 227, and *Aspergillus niger* MTCC 282. The cell lines were purchased from NCCS, Pune, India.

### Chemicals and media.

All chemicals and solvents were of analytical grade and purchased from Merck, Germany and culture media were obtained from Hi-media, Mumbai, India. Standard doxorubicin was obtained from Sigma-Aldrich.

### Collection of soil samples.

Soil samples were collected at depths of 3–5 cm below the surface from various forest areas in and around Nagpur, India. The samples were placed in sterile polyethylene bags, closed tightly and stored at 4°C until required.

### Treatment of soil samples and selective isolation of rare actinomycetes.

Approximately 1 g of each sample was suspended in 10 ml sterile distilled water from which three dilutions (10^−2^ to 10^−4^) were prepared. The diluted samples were divided into equal aliquots, and were subjected to various treatments like 1.5% phenol treatment, 0.3% Chloramine T treatment, benzethonium chloride treatment, heat treatment, air drying and a combination of these treatments before plating on appropriate isolation media (9). Several media such as starch casein agar, humic acid vitamin agar (HVA), actinomycetes isolation agar (AIA), yeast extract-malt extract-dextrose agar (ISP2), glycerol-asparagine agar (ISP5), and tyrosine agar (ISP7) were used for isolation (10, 11). All media were supplemented with sterile antifungal (cycloheximide 100 μg/mL, nystatin 25 μg/mL) and antibacterial (gentamicin, vancomycin, streptomycin, and nalidixic acid 25 μg/mL) antibiotics to facilitate the selective isolation of slow-growing rare actinomycete genera.

Inoculated plates were incubated at 30°C for up to six weeks, and all leathery colonies were identified and sub-cultured on starch casein agar. Preliminary identification of rare actinomycete colonies were done by microscopic observation with a long working distance microscope. Single colonies were successively transferred onto potato dextrose agar and incubated until pure isolates were obtained.

### Characterization of non-streptomycete actinomycetes.

The growth of rare actinomycete cultures was examined at 7, 14 and 21 days on ISP2 and starch casein agar. The presence of aerial mycelium, the color of aerial and substrate mycelium and the formation of soluble pigments were recorded. Cover slip culture method was used for the microscopic characterization. The mycelium structure, arrangement of conidiospores and arthrospores on the mycelium were observed by high power (400X). The diaminopimelic acid (DAP) isomer in the cell wall was determined by the method of Becker et al. (12). The whole cell sugar pattern (WCSP) was obtained by the method of Staneck and Roberts (13). Lysozyme sensitivity of the isolates was determined to differentiate between Streptomycetes and non-streptomycetes (14).

### Screening for bioactivity.

The rare actinomycete isolates were assessed for their capability of producing bioactive compounds by agar cross streak method against test organisms (15). Isolates that showed activity in the primary screening were selected for secondary screening. These isolates were grown in submerged culture in 250 ml flasks containing 50 ml of PDB (Potato Dextrose Broth) medium. The cell free supernatant, sterilized by filtration and vacuum concentrated five-fold in Vacufuge Plus (Eppendorf, North America) was used for extracellular antimicrobial activity by agar well diffusion method against test microorganisms (16).

### Anti-Quorum sensing activity.

For the anti-QS screening, the rare actinomycetes were first central streaked onto the PDA plates and incubated for 2–3 days. The plate was then overlaid with soft LB agar seeded with the indicator strain *Serratia marcescens* and incubated overnight. A positive QSI result was indicated by a lack of pigmentation of the indicator strain around the vicinity of the test organisms. Negative results were indicated by no pigmentation inhibition (17).

### PCR detection of PKS-I, PKS-II, and NRPS sequences.

Three sets of degenerate primers were used for the amplification of genes encoding polyketide synthases I and II (PKS-I and PKS-II) and nonribosomal peptide synthetases (NRPS) (Table 1) (6, 18). The PCR reaction mixture that consisted of 20–200 ng genomic DNA, 10.0 μL of 2X GoTaq Green master mix (Promega, USA), 10 pmoles of different primer sets, and sterile ultrapure water was added to final volume of 20 μL. The PCR was performed using the Bio-Rad thermal cycler with the following cycling conditions: (i) 94°C for 5 min; (ii) 30 cycles of 94°C for 1 min, 57°C (for K1F-M6R and KSα/KSβ) or 62°C (for A3F-A7R) for 1 min, and 72°C for 2 min; and (iii) 72°C for 5 min. The PCR amplification products were resolved using electrophoresis in 1% agarose gel (Promega, USA) and stained with ethidium bromide (0.5 μgmL^−1^) and viewed using Molecular Imager. *Streptomyces nogalater* was used as a reference strain for the presence of PKSII gene and *Streptomyces avermitilis* for NRPS and PKS I genes.

**Table 1:** PCR primers used in this study

**Primers**	**Primer Sequence**	**Gene recognized**	**Product size (bp)**	**Reference**
K1F	TSAAGTCSAACATCGGBCA	PKS I	1200	6
M6R	CGCAGGTTSCSGTACCAGTA			
KSα	TSGCSTGCTTCGAYGCSATC	PKS II	613	18
KSβ	TGGAANCCGCCGAABCCGCT			
A3F	GCSTACSYSATSTACACSTCSGG	NRPS	700	6
A7R	SASGTCVCCSGTSCGGTAS			
27F	AGAGTTTGATCMTGGCTCAG	16S rRNA	1400	45
1492R	AGAGTTTGATCMTGGCTCAG			

### DNA extraction, sequencing and analysis.

Total genomic DNA from seven potent isolates was extracted using standard method (19). The 16S rRNA gene was amplified by PCR using the primer pair 27F-1492R (Table 1). PCR was carried out under the following conditions: initial denaturation at 94°C for 3 min; 30 cycles of 94°C for 30 sec, 60°C for 30 sec, and 72°C for 1 min; and a final extension at 72°C for 10 min. The PCR products were purified and sequenced using a ABI PRISM® BigDyeTM Terminator Cycle Sequencing Kits with AmpliTaq® DNA polymerase (FS enzyme) (Applied Biosystems).

The resultant 16S rRNA gene sequences were compared with those of other closely related taxa retrieved from the GenBank database. The program MUSCLE 3.7 was used for multiple alignments of sequences (20). The resulting aligned sequences were cured using the program Gblocks 0.91b. Finally, the program PhyML 3.0 aLRT was used for phylogeny analysis and HKY85 as substitution model. The program Tree Dyn 198.3 was used for tree rendering (21).

### Physico-chemical screening.

The absorption spectra of bioactive crude organic extracts in methanol were recorded in the UV-visible region (190–1100 nm) by using a biospectrophotometer (Model BL 198, Elico, India) and observed for peaks in 230–280 nm range and 400–500 nm range for detection of aromatic polyketides (22), 215–270 nm for complex polyketides like polyenes compounds (7) and in 210–230 and 270–280 for peptides (23). Doxorubicin, an anthracycline; nystatin, a polyene and nisin, a peptide were used as standards.

Chemical screening of the crude extracts was carried out by the method described by Taddei (8). TLC screening was performed on readymade silica gel plates (Merck, Germany, Cat # 1.05554.0007). All samples were spotted manually with a 5-μl micro-pipette. Each TLC plate (20 × 20 cm) was spotted with samples of the various microbial crude extracts and the chromatograms were developed using chloroform: methanol: aq ammonia (85:14:1), or benzene: acetone: methanol (100:10:1), methanol: chloroform: acetic acid: water (80:20:2:0.2) chloroform: heptane: methanol (10:10:3) as the solvent systems.

Detection of secondary metabolite patterns was performed using a UV lamp at 254 and 365 nm, as well as by direct staining using coloring reagents like magnesium acetate reagent, formaldehyde- H_2_SO_4_ reagent, naphthoresorcinol reagent and chlorine o-dianisidine (8, 23).

### Anticancer activity.

The effect of bioactive extracts on HeLa was determined *in vitro* by MTT assay (24). The adherent cells were exposed to a concentration gradient of 1–10 μg/ml.

## RESULTS

### Effect of pretreatment & antibiotics for selective isolation of rare actinomycetes.

Using different soil treatment methods, selective media and cultivation conditions, organisms putatively identified as actinobacteria were selected on the basis of characteristic colony morphology, notably the ability to form aerial hyphae and substrate mycelia.

1.5% phenol treatment of soil suspension yielded the maximum number of colonies, sixteen, on ISP5 supplemented with gentamicin followed by ISP7 with the same antibiotic. Air dried samples gave maximum colonies, nine, on starch casein agar (SCA) supplemented with gentamicin followed by five colonies on ISP 7 with streptomycin. With heat treatment, AIA, ISP5 and ISP7 with vancomycin yielded three, five and two colonies. However, maximum colonies nine, were obtained on ISP2 with gentamicin. Chloramine treatment was found to be most successful on ISP7 with vancomycin and nalidixic acid (Table 2).

**Table 2:** Recovery of rare actinomycetes colonies using selective isolation methods

**Treatment**	**Media**	**Antibiotics used**	**No. of colonies**	**Reference**
1.5% phenol	ISP 5	Gentamicin	16	46
1.5% phenol	ISP 7	Nalidixic acid/gentamicin	10	47
Air dry	SCA	Gentamicin	9	48
Air dry	ISP 7	Streptomycin	5	49
Heat treatment	ISP 2	Gentamicin	7	50
Heat treatment	ISP 5	Kanamycin	5	51
Chloramine treatment	ISP 7	Streptomycin	4	52

A total of one hundred and seventeen actinobacterial strains were isolated from five forest soil samples and one hundred and six isolates were found to have meso-DAP indicating that they are rare actinomycetes.

### Identification of rare actinomycetes.

The isolates were grouped on the basis of colour series, the main colours being white (45.83%), gray (35.41%), red (6.25%), and yellow (4.16%); while 8.33% isolates were without any distinct colour (hyaline). Whole cell sugar pattern of isolates containing meso-DAP showed that fifty strains had xylose either alone or with arabinose. Eighteen strains showed the presence of madurose and thirty two, galactose. Using a polyphasic approach, on the basis of phenotypic, microscopic and chemotaxonomic criteria, the isolates were classified into twelve genera as shown in Table 3. The isolates were renamed as per their generic name for further studies.

**Table 3:** Grouping of isolates into genera on the basis of morphological and chemotaxonomic properties

**Genus**	**No. of isolates (%)**	**Cell wall amino acid**	**Cell wall type**	**Whole cell sugar pattern**	**Substrate mycelium**	**Aerial mycelim**	**Spores**
*Micromonospora*	22 (20.75)	Meso- DAP, glycine	II	Ara, Xyl (D)	Yellow/brown	-	Spherical to oval
*Dactylosporangium*	16 (15.09)	Meso- DAP, glycine	II	Ara, Xyl (D)	Yellow brown/orange	-	Single row of 3–4 oblong spores
*Actinoplanes*	12 (11.32)	Meso- DAP, glycine	II	Ara, Xyl (D)	Orange/yellow	-	Spherical or short rods
*Nocardiopsis*	11 (10.3)	Meso-DAP	III	No (C)	Creamish yellow	Grayish white	Long chain of spores
*Actinomadura*	10 (9.43)	Meso-DAP	III	No (C)	Grayish white	white	Short chain of conidia on aerial mycelium, often curled
*Microbispora*	9 (8.49)	Meso-DAP	III	Ara, Gal, Mad	Yellow/orange	Pink white	Chains of conidia with 2 spores
*Catellatospora*	7 (6.6)			Ara, Xyl, Gal	Bright yellow	No	Short chain of conidia
*Planobispora*	6 (5.66)	Meso-DAP	IV	Ara, Gal, Mad (B)	Brownish white	White with rose tinge	Spornagia with 2 spores
*Kocuria*	5 (4.71)					-	
*Spirilliplanes*	4 (3.77)	Meso -DAP		Xyl, Gal, Mann	Yellow/orange	white	Oval or short rods
*Spirillospora*	3 (2.83)	Meso -DAP	III	Mad (B)	yellow	white	Rod shaped or curved
*Saccharomonospora*	1 (0.94)	Meso-DAP	III	Ara, Gal (A)	white	Grayish green	Spores in pairs or shortchains

Seven potent isolates were identified on the basis of 16S rRNA gene sequencing. The sequences have been submitted in Genbank and accession numbers obtained as shown in Table 4.

**Table 4:** Similarity of the rare actinobacterial isolates to the closest cultivated species and their Genbank accession numbers

**Isolate**	**Genbank accession No.**	**No. of nucleotides sequenced**	**Query cover**	**Similarity**	**Closest cultivated species (GenBank accession no.)**
ACM1	MG372011	1167	100%	79%	*Actinobaculum schaalii* strain NML 070171 16S ribosomal RNA gene, partial Sequence (FJ711188.1)
KCR3	MG430204	938	100	100	*Kocuria kristinae* partial 16S rRNA gene, strain SZ22 (LT600550.1)
ATP10	MG388286	1163	100%	79%	*Actinomyces* sp. VUL8 16S ribosomal RNA gene, partial sequence (KX389558.1)
MMS16	MG372012	1203	94%	81%	*Micromonosporaceae* 16S rRNA gene, isolate SR 53 (X87321.1)
MBS9	MG388285	1191	100%	78%	Actinomycetales bacterium AB1007 16S ribosomal RNA gene, partial sequence (JQ924089.1)
ACM9		1427	100	99%	*Glutamicibacter arilaitensis* strain Re11716S rRNAgene, partial sequence (NR074608.1)
MMS8	MG407702	1143	96	98%	*Micromonospora auratinigra* strain RLFI_1037 16S rRNA gene, partial sequence (KY580811.1)

### Primary screening.

When the isolates were subjected to primary screening using agar cross streak method, ninety eight isolates showed antimicrobial activity against one or the other test organism. Sixty two isolates showed only antibacterial activity, twelve isolates showed only antifungal activity and the remaining twenty four isolates showed both antibacterial and antifungal activity. Of the isolates with antibacterial activity, fifty isolates were active only against Gram positive bacteria whereas twelve isolates were active against both Gram positive as well as Gram negative bacteria. Thirty seven (36.2%) isolates showed anti-quorum sensing activity when tested against *Serratia marcescens*.

### Secondary screening.

All the ninety eight bioactive isolates selected in primary screening were subjected to secondary screening against *B. subtilis, E. coli* and *C. albicans*. Only ninety one isolates showed antibacterial or antifungal activity in secondary screening. The extracts of fourteen isolates were observed to have a remarkable antibacterial activity of which isolate MMS17 was found to be the most potent with a zone of inhibition of 7.3 cm against *B. subtilis* and 4.5 cm against *E. coli*. Similarly, the extracts of ten isolates showed remarkable antifungal activity, with isolates SMS and MMS16 being more active against *C. albicans* and isolate DCT11 and ATP10 against *A. niger*. Of the eleven isolates which showed anti QS activity against *S. marcescens*, five isolates namely, isolate KCR3, MMS14, MMS16, SMS and NCD10 showed remarkable activity. Two isolates, isolate MMS16 and SMS exhibited all the three activities that is, antibacterial, antifungal and anti QS activities.

### Detection of PKSI, PKSII & NRPS genes.

A total of eighty four (92.3%) strains were found to possess at least one of the PKS and NRPS systems. NRPS sequences were detected in thirty nine isolates (42.8%), whereas PKS-I and PKS-II sequences were detected in seventeen and twenty eight strains (18.6% and 30.7%), respectively. Thirty six isolates showed the presence of two genes. All three target genes were detected in five isolates (MMS1, MMS9, MMS14, DCT14, MMS16).

### Physicochemical screening.

Out of the twenty eight bioactive isolates which also showed the presence of the PKS II gene, the extracts of twenty one isolates showed UV absorption maxima in the range 208 and 288 nm indicating the presence of an aromatic nucleus. When the extracts of these twenty one isolates were chemically screened, nineteen showed a color reaction with formaldehyde-sulphuric acid reagent confirming the presence of an aromatic nucleus in their structure. The UV-Visible spectra of at least seventeen extracts showed an additional peak in the 400–500 nm range, which could indicate the presence of an extended chromophore as seen in aromatic polyketides. Chemical screening using naphthoresorcinol reagent showed the presence of sugars in the extracts of these seventeen isolates. Of the seventeen isolates which showed amplification of PKSI gene, the extracts of nine isolates showed characteristic peaks in the range of 215–270 indicating their capability of producing type I polyketides.

Results of physicochemical screening using absorption measurement at 254 nm and 366 nm and reaction with chlorine o-dianisidine showed that twenty four isolates could be recognized as peptide producing strains. None of the spots reacted with ninhydrin reagent. The UV spectral data for the ethyl acetate extracts of selected active fermented broth, antimicrobial activity and presence or absence of biosynthetic genes are shown in Table 5.

**Table 5:** Summary of culturable rare actinomycete isolates from Indian forest soils

**Isolates**	**Aerial mycelim**	**Diffusible pigment**	**λ max**	**Antimicrobial activity^a^**			**Anti QS activity**	**Biosynthetic genes^b^**			

				***B. subtilis***	***E. coli***	***C. albicans***	***S. marscenes***	**PKS I**	**PKS II**	**NRPS**
ACM7	Yellow	Yellow	235, 377, 420	+	+	+	+	-	-	-
MMS6	Orange		241, 380, 445	+	+	-	-	-	-	-
MMS8	Orange	Orange	252, 317, 460	++	+	+	+	-	+	+
MMS9	Pale yellow		291, 299, 362	++	+	+	-	+	+	+
MMS10	White		217, 289	++	-	-	+	+	-	+
KCR3	Pale orange		280,293	+	-	+	+++	+	-	+
MMS13	White		303, 330	-	-	-	+	-	+	+
MBS6	Yellow		342, 358, 377,	++	-	-	+			
MMS14	Orange	Orange	266, 276	+	-	-	+++	+	+	+
MMS15	Pale yellow		288, 342, 378, 405	++	-	+	+++	+	+	-
MMS16	Yellowish orange	Yellow	352, 363, 377,	+++	++	++	+++	+	-	-
SMS	White		337, 345	+++	++	+++	+++	-	-	+
ATP6	White		338, 377, 417, 438	+	-	-		-	+	-
MMS17	White		340, 377, 420, 445	+++	++	-	+	+	+	-
DCT11	Reddish brown		296, 351	+++	++	-	+	+	+	-
ACM9	yellow		347, 333, 358, 378	-	-	++	-	+	+	-
ATP10	Yellowish orange		347, 437	+	-	++	-	-	+	+
DCT12	Pale yellow		327, 400	+	-	-	-	+	-	-
MMS20	Dark yellow		363, 375, 417, 438	+	+	+	-	+	+	-
MBS8	Orange	Yellowish	317, 343, 362,	-	-	++	-	-	+	+
MMS22	White		242, 352	-	-	+	-	-	-	-
DCT14	White		335, 340, 375, 403	+	-	-	++	+	+	+
DCT15	Bright orange		342, 377, 333	-	+	-	-	+	-	-
MMS21	White		317, 332, 342	+	+	-	-	+	-	-
MBS9	Yellow		330, 358, 412	++	++	-	++	-	+	-
NCD10	Yellow		291	++	+	+	+++	-	-	+
NCD11	White		338, 358, 400	++	+	-	++	+	+	-

a +++ inhibition zone >15mm, ++ inhibition zone 5–15mm, + inhibition zone 0–5mm, - No inhibition

b + Present, - Absent

### Anticancer activity.

On the basis of potency and diversity of bioactivity, the extracts of eight isolates (KCR3, MMS14, MMS16, SMS, MMS17, DCT14, MBS9, NCD10) were tested for anticancer activity against HeLa cell line using MTT assay. The extracts of isolates KCR3 and NCD10 were found to be the most potent with IC_50_ value of 3 μg/ml and 2.5 μg/ml respectively and other extracts in the range of 4.5 to 6.5 μg/ml.

## DISCUSSION

A large number of actinomycetes have been isolated and screened from soil in the past several decades, accounting for 70–80% of secondary metabolites available commercially (25). Consequently, the possibility of isolating new actinomycete strains has diminished so that the search for novel products has now switched to rarer genera of actinomycetes.

### Selective isolation of rare actinomycetes.

Non-Streptomycete actinobacteria can be isolated using chemical and physical pretreatment of the samples, specific selective media, fine-tuning of culture conditions and other genus-specific methodologies (9), including the use of anti-Streptomyces antibiotics (Table 2). Streptomycin or nalidixic acid and kanamycin in combination have been used for isolation of *Actinomadura* by Hayakawa and others (10, 26). Gentamicin has been specifically used for that of *Dactylosporangium* previously (27).

In this study, 1.5% phenol treatment was found to be the most successful method resulting in isolation of almost 40% of the total rare actinomycetes. Four genera, *Micromonospora, Actinoplanes, Actinomadura* and *Microbispora*, that could resist the germicidal effect of phenol were isolated by this method. Our results were in agreement with that of Hayakawa et al. and Istianto et al. (28, 29).

Genera *Micromonospora, Actinoplanes, Actinomadura* and *Saccharomonospora* were recovered by using dry heat pretreatment. Similar results were obtained by Hayakawa and Kavitha et al. (9, 30), who showed that heating air dried soil samples at 120°C reduced the numbers of filamentous bacteria and streptomycetes on isolation plates, resulting in the selective isolation of various rare actinomycetes genera.

In this study, *Micromonospora* was the most abundant genus followed by *Dactylosporangium. Actinomadura, Nocardiopsis* and *Actinoplanes* were almost equal in number. Jose and Jebakumar have reported *Micromonospora* as the second most dominant group after *Streptomyces* accounting for 23% of total actinomycetes in their study (31). *Micromonospora* was also the major genera of rare actinomycetes in both mangrove and soggy soils (32). In Indian scenario too, research on actinobacterial diversity from terrestrial ecosystems has led to the isolation of *Micromonospora, Actinomadura* and *Microbispora* (33).

### Bioactivity.

Our results using cross streak method indicated that almost 92% of the isolates were active against one or more of the test organisms which was much higher than that reported by Qin et al. and Lee et al. (3, 34).

Almost 26.5% of the bioactive isolates, in this study, were found to be broad spectrum showing both antibacterial and antifungal activity. Most of the antibacterial isolates were found to inhibit only the Gram-positive bacteria (81.2%). Many authors working on bioactive actinomycetes have reported similar results (3, 35).

A few isolates (7.14%) which showed activity in primary screening did not show activity in secondary screening. This is in line with results reported earlier by some researchers (36). In this screening project, we found that thirty seven isolates obtained from soil showed anti-QS activity against *Serratia marcescens.* We followed the ‘soft agar overlay protocol’ developed by McLean et al. based on pigmentation inhibition to rapidly screen for the presence of potential QSI by rare actinomycete isolates (37). However, in our assay, instead of *Chromobacterium violaceum* we used *Serratia marcescens*, which produces a red pigment controlled by AHL-mediated QS systems, as an indicator organism for the screening of QSI (17).

### Detection of PKSI, PKSII & NRPS genes.

The bioactive actinomycete isolates from soil samples were screened using degenerate primers for the presence of PKS-I, PKS-II and NRPS genes. NRPS sequences were detected in 42.8% isolates, which is similar to that reported by Yuan et al. but more than Qin et al. and Lee et al. and less as compared to that of 68% reported by Pathom Aree et al. In this study, PKS I sequences were detected in 18.6% which is in agreement with that of Qin et al., Lee et al. and Pathom Aree et al., but much less than that reported by Yuan et al. Also, PKSII detection was observed in 30.7% isolates, slightly lower than that found by Yuan et al. and Lee et al. but higher than Qin et al. (3, 34, 35, 38).

Some isolates such as MMS9, KCR3, MMS16, MMS17, DCT14, and MBS9 exhibited antibacterial activity against test organisms and these correlated well with successful amplification of either one or more of the targeted genes from the genomes of the isolates. It can be inferred that these isolates contain at least one complete biosynthetic gene cluster for bioactive secondary metabolite production.

In this study, some actinomycete isolates such as ACM7, MMS6, DCT11, MMS22 and NCD9 exhibited antimicrobial activity but no amplification products. The absence of PCR amplicons in these isolates suggests the lack of the biosynthetic genes although this assumption can not be conclusive. According to Wood et al. (39) this might be as a result of variations in primer target sequences preventing the primers from binding efficiently. A limitation in this kind of study is that the design of degenerative primers is based on the available sequences in the GenBank of more abundant and well-studied organisms such as *Streptomyces*, unlike the rare actinomycetes that have few available sequences on their biosynthetic gene clusters in database library. Some isolates such as MMS12, DCT7, DCT13 and CTS5 did not exhibit activity but there was amplification of the biosynthetic gene cluster. Not all bio-synthetic gene clusters are involved in the biosyn-thesis of bioactive secondary metabolites (40, 41). It is also possible that the genes detected by PCR are non-functional or the isolates in question might have different nutritional requirements for the production of bioactive secondary metabolites (3). Also, Wood et al. concluded that positive PCR amplification does not indicate that the genes are expressed nor does it show that the strain possesses the full suite of biosynthetic gene cluster for the biosynthesis of that class of antibiotic. The detection of all the three genes in some isolates such as MMS1, MMS9 and DCT14 are indicators of their potential natural product diversity and divergent genetic evolution.

### Physicochemical screening.

Physico chemical screening using UV-visible spectroscopy and TLC was used to detect the ability to produce polyketides and peptides by rare actinomycetes. A λ max of 417 nm, 447 nm and 532 nm indicates the polyketide nature of the bioactive compounds. An additional peak of 288 nm is indicative of an aromatic nucleus in the structure indicating that the polyketides may be Type II polyketides (22). UV-visible spectral studies have been used to predict the nature of compounds by comparing data with available literature. On this basis isolates MMS8, MMS10, MBS6, MMS16, ATP6, ATP10, MMS20, MBS8 and MBS9 were found to be aromatic polyketide producers. Almost all the types of aromatic polyketides contain sugars attached to their chromophore in their structure (42). Thus the presence of sugars in the metabolite fingerprinting of these isolates indicates that they might be potential aromatic polyketide producing species. Also the extracts of isolates MMS8, MMS10, MBS6, MMS16, ATP6 and MBS9 were found to be pH sensitive further confirming that they may be anthracyclines, a class of type II polyketides (22). The extracts of ATP10, MMS20 and MBS8 were not pH sensitive indicating that they can be other type II polyketides like tetracenomycins or tetracyclines. Except isolate MMS10, in all these isolates the amplification of gene for PKS II was also observed.

Many researchers have used this technique to screen organisms for polyenic antifungal compounds, which are type I polyketides (7). The spectral data of the extracts of isolates MMS9, MMS13, MMS15, DCT11, ACM9, DCT12 and DCT15, MMS21 and NCD11 was consistent with those obtained by Maleki et al. and Ilic et al. (43, 44). UV spectral data of the extracts of isolates KCR3, MMS14, SMS, MMS17, PBS4 and NCD10 exhibited strong absorption maxima at 250–270 nm, which corresponds to the characteristic absorption of a peptide bond. A peptide antibiotic cerein, obtained from *B. cereus* shows UV absorption maxima at 198, 255 and 273 nm and weak absorbance peak at 250 and 273 nm. Further, chlorine o-dianisidine that reacts with peptides was used to find peptide-producing strains. Positive reaction of TLC spots of extracts of isolates KCR3, MMS14, SMS, MMS17 and NCD10 with chlorine o-dianisi-dine reagent suggested they are peptides and the absence of coloration of the TLC spots with ninhydrin further suggested that they are cyclic (23).

## CONCLUSION

This study led to the isolation of nine strains each of type I and type II polyketide producers, and six strains of peptide producers. The combined strategy of molecular and physicochemical screening along with bioactivity guided screening was found to be an effective approach in looking for polyketide and peptide producing rare actiomyceytes. The extract of isolate KCR3, a peptide and that of isolate NCD10, a polyketide was found to possess anti tumor activity exhibiting an IC_50_ value of 3 μg/ml and 2.5 μg/ml against HeLa cells. These compounds can serve as potential candidates for development as chemotherapeutic drugs for the future.
